# Translating ‘See-and-Treat’ to primary care: opening the gates does not cause a flood

**DOI:** 10.1093/intqhc/mzy244

**Published:** 2019-01-09

**Authors:** Carl Savage, Staffan Bjessmo, Oleg Borisenko, Henrik Larsson, Jacob Karlsson, Pamela Mazzocato

**Affiliations:** Department of Learning, Informatics, Management and Ethics, Medical Management Centre, Karolinska Institutet, Stockholm, Sweden

**Keywords:** See-and-treat, primary care, quality improvement, standardization, patient engagement

## Abstract

**Objective:**

To explore how the See-and-Treat concept can be applied in primary care and its effect on volume and productivity.

**Design:**

An explanatory single-case study design with a mixed methods approach and presented according to the SQUIRE 2.0 guidelines.

**Setting:**

A publicly-funded, private primary care provider within the Stockholm County, which caters to a diverse patient population in terms of ethnicity, religion, socioeconomic status and care needs.

**Participants:**

CEO, center manager, four physicians, two licensed practical nurses, one medical secretary and one lab assistant.

**Intervention:**

A See-and-Treat unit was established to offer same-day service for acute unplanned visits. Standardized patient symptom forms were created that allowed patients to self-triage and then enter into a streamlined care process consisting of a quick diagnostic lab and a physician visit.

**Main Outcome Measures:**

Volume, productivity, staff perceptions and patient satisfaction were measured through data on number and type of contacts per 1000 listed patients, visits per physician, observations, interviews and a questionnaire.

**Results:**

A significant decrease in the acute and total number of visits, a continued trend of diminishing telephone contacts, and a non-significant increase in physician productivity. Patients were very satisfied, and staff perceived an improved quality of care.

**Conclusions:**

See-and-Treat appears to be a viable approach for a specific primary care patient segment interested in acute same-day-service. Opening up access and standardizing care made it possible to efficiently address these needs and engage patients.

## Introduction

Primary healthcare centers (PHCC) are designed to be patients’ first point of access for non-urgent, chronic and preventive care services. This role encompasses responsibility for the provision of accessible, continued, comprehensive and coordinated care [1]. Despite this well-defined role, long waiting times hinder access to care [2]. This can lead to the inefficient use of other health system entry points [3] such as emergency departments (EDs), which can lead to overcrowding and risk patient safety.

Walk-In Centers (WIC) and Retail Clinics (RC) were developed to improve access to primary care and serve as alternatives to EDs in the UK and the USA, respectively. Neither requires an appointment, are usually led by nurse practitioners, operate at convenient locations with extended hours which is why patients choose them, and provide immediate and episodic care for treatment of minor illnesses and injuries [4–7]. Patients are highly satisfied [8], care quality is comparable to PHCCs [9], but they can increase demand or lead to duplication of services [4, 10] and negatively impact care continuity [11–13]. It has been suggested that the inclusion of physicians could increase the benefits [14].

EDs are also challenged by waiting times and overcrowding [15, 16], and solutions developed there may be applicable to primary care. See-and-Treat is an approach to flow improvement by assigning staff to a separate stream for patients with low acuity conditions. It has reduced waiting time for these patients as well as for the ED in general [17].

The development of processes tailored to meet the needs of patients with less severe conditions, but which require physician-level competence, may expand the scope and effectiveness of WIC and RCs. As with EDs, a See-and-Treat could potentially benefit the entire PHCC. Thus, the aim of this study was to explore how the See-and-Treat concept can be applied in primary care and its effect on volume and productivity.

## Methods

### Study of the intervention

This explanatory single-case study using mixed methods [18] follows the SQUIRE 2.0 guidelines ‘for quality improvement reporting excellence’ [19]. It represents a unique translation of See-and-Treat to primary care.

### Context of the intervention

Swedish healthcare is public, single-payer and tax-based. Providers are predominantly public, but care can also be procured from private providers. One such publicly-funded, privately-owned provider founded a PHCC in a suburb of Stockholm, Sweden in September 2010. Inhabitants represented a diverse population in terms of ethnicity, religion, socioeconomic status and care needs. Within three years, 10 000 patients had listed themselves. The increase negatively impacted care access, patient and staff satisfaction, productivity and profit margins.

Patients called to book visits or consult with a nurse (RN). Failure to answer within 1 min incurred a financial penalty, so three RNs were assigned to telephones. Fifteen-minute time-slots were reserved for acute same-day physician consultations. With too many patients, the practice was to double-book patients or refer them to other caregivers. This incurred penalties and lowered patient satisfaction. The typical care process involved two to four professionals: receptionist, physician, laboratory assistant and nurse. Tests were conducted in an on-premises lab. The waiting room was crowded, and delays frustrated physicians. Temp agencies were used frequently. Because space and economic limitations prevented the hiring of more physicians, management wanted to increase productivity with existing staff.

### The See-and-Treat intervention

One physician recognized that the time-slot approach impaired physicians’ ability to see more patients if a visit took <15 min. The idea emerged to streamline unplanned visits through dedicated lab resources and patient self-triage. In consultation with the physician group, the sixteen most frequent presenting complaints were identified. After reviewing their own procedures for these complaints, standardized strategies for clinical examination were developed, discussed and internally validated by the physicians.

Each standardized strategy was summarized in a paper ‘symptom form’ consisting of two fields: an upper, with questions to be answered by the patient, and a lower checklist with tests, diagnoses, and treatments for the lab and physician, with space for comments. With management support, a dedicated ‘acute’ lab with tests that could be performed within 2 min was created in a rebuilt storage closet and staffed with a licensed practical nurse (LPN). Two examination rooms, a sitting area and a separate entrance were eventually appropriated. Pilot-testing with patients began in October 2013. Opening hours and days were stepwise increased until full implementation began in February 2014. Key principles were:

- All patients that come are welcome.
- Every patient should meet a physician the same day he/she needs help.
- A standardized form specific to a patient’s symptoms is used to collect patient and diagnostic information and forms the basis for documentation.
- Staff and facilities are dedicated to the See-and-Treat
- Patients with complex needs are referred to the other part of the PHCC after their visit.

### Measures

Multiple data sources were used. Ten semi-structured interviews were conducted using a pilot-tested interview guide that explored the content and context of the intervention, its evolution and staff perceptions. Participants (50% women) included the CEO of the parent company, the PHCC manager, four physicians, two LPNs, one medical secretary and one lab assistant. Interviews were conducted at participants’ workplaces, lasted ~1 h, were digitally recorded, and transcribed *verbatim*. Based on the interviews, we designed an observation protocol to map and observe care processes over four days (*n* = 86 patients). Fourteen additional patients were observed in critical segments of the process to validate the analysis. Capacity data on physicians and listed patients were collected through administrative systems. Symptom forms provided information on presenting complaints.

Data on volume and productivity were collected from the electronic health records (EHR) (weekdays from January 2013 to March 2015). Changes in volume were measured by calculating the total number of visits, acute visits and telephone calls per 1000 listed patients. Since See-and-Treat patients could not be separated from unplanned visits scheduled as same-day acute visits in the EHR, we included all acute visits. Total physician productivity was calculated as the total number of visits per physician per 1000 listed patients.

Patient satisfaction was measured with a paper-based questionnaire consisting of seven questions about the experience with a five-point Likert scale. All patients during a three-week period one month after the intervention began were asked, upon the completion of their visit, to fill it out and leave it with the LPN.

### Analysis

Interviews were analyzed with conventional content analysis [20]. Meaning units related to content (i.e. the process steps and facility redesign), influential contextual factors, and how the process evolved were identified and coded [21]. Observational data, which was analyzed to identify key activities, decision points, and resources utilized, helped refine and validate process maps.

Patient characteristics and satisfaction data were analyzed descriptively. Volume and productivity were analyzed quarterly (four time points before and after intervention). The time of intervention was set at February 2014 (first quarter of 2014 in the analysis). Data about the proportion of acute visits were not available for all months of observation; imputation using the same month’s data was used when necessary. We controlled for changes in volume and productivity not related to the intervention by applying interrupted time-series (ITS) analysis using autoregressive integrated moving average (ARIMA) and time-series regression techniques for data obtained only from the period 13 months before and after implementation [22]. ITS controls for secular trend and autocorrelation. Statistical analysis was performed using SPSS version 20 (IBM Corp., Armonk, New York, USA) with Data Forecasting functions. Due to the limited number of observations available, seasonal decomposition was not possible in SPSS and was performed in Microsoft Excel 2010 (Microsoft Corp., Redmond, Washington, USA).

### Ethical considerations

Interviewees and questionnaire respondents were informed that participation was voluntary and that they could withdraw at any time. Informed consent was obtained prior to the interviews. Data were treated to ensure confidentiality and anonymity. No personal data on patients was collected. Ethical vetting was obtained from the Stockholm Regional Ethics Committee (2014/1304–31).

## Results

### The intervention and its evolution

Patients calling to book same-day appointments are informed through an answering machine of the See-and-Treat option. At the PHCC, signs direct patients to a separate See-and-Treat entrance. In the waiting area, a sign instructs patients to take a queue number and select and fill out the most appropriate of sixteen symptom forms from a wall display. An LPN registers the patient, accepts payment, and chooses the relevant lab after a doctor had been consulted (if needed). The form with the lab results follows the patient into the examination room. The physician verifies the symptoms and performs a semi-standardized clinical examination with all the instruments set-up within arm’s reach. A nurse follow-up is booked if needed. In total, the patients meet two to three professionals: the LPN, the physician, and sometimes a nurse. A medical secretary scanned the forms into the EHR. Based on observational data, average time with the physician was ~6 min, 12 s (*s* = 4 min 11 s) and total door-to-door time was ~28 min.

In 3.5% of patients, additional laboratory tests were needed. If the lab became a bottleneck, some patients (5%) were sent to the main lab. The LPN was eventually replaced by an RN to raise the competency level. To improve reporting quality, physicians began to dictate their notes, but after complaints from the medical secretary about the increased workload, physicians began to type directly into the EHR.

Signage proved inadequate; the LPN instructed 38% of patients about how to correctly choose and fill out the symptom form. Signage was therefore increased and redesigned. The most common complaints were adults or parents with children presenting with upper airway infections (39%), sore throats (13%), dermatological problems (11%), back pain (10%), lower urinary tract infections (9%), ear pain (9%), abdominal symptoms (4%), eye conditions (3%), headaches (2%) or for a prescription renewal (1%).

Opening hours were increased from two mornings/week to weekday mornings and afternoons. On average, 5.4 physicians were on duty at the PHCC with one working at the See-and-Treat. During the morning rush (08.30–10.00), an additional physician could be called in.

### Effects of the intervention

#### Effects on volume and productivity

Between January 2013 and March 2015, physician visits numbered 49 260, of which 33.5% (*n* = 16 496) were unplanned acute visits. See-and-Treat patients increased continuously and eventually stabilized at an average of 33 per day (*s* = 9.0). In total, 73 945 telephone calls were answered. Listed patients increased by 26% (9072–11 404).

**Figure 1:**
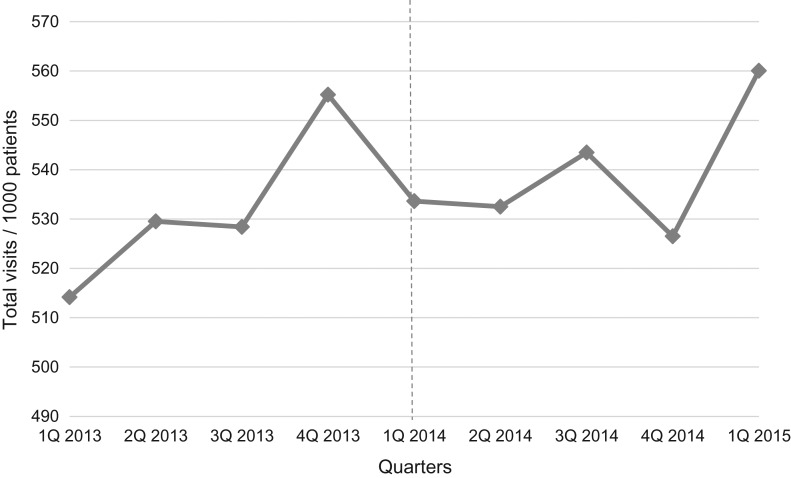
Total number of visits per 1000 listed patients from the first quarter of 2013 to the first quarter of 2015 (after adjustment for seasonality). Dotted vertical line indicates time of implementation of the See-and-Treat.

**Figure 2:**
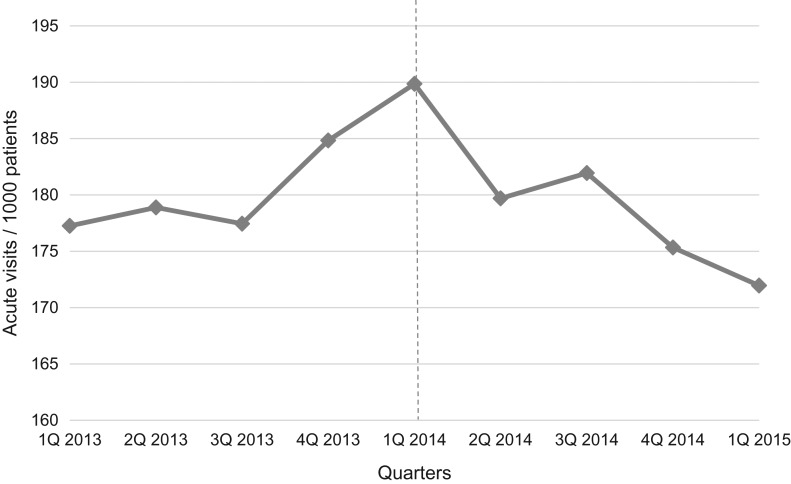
Number of acute visits per 1000 listed patients from the first quarter of 2013 to the first quarter of 2015 (after adjustment for seasonality). Dotted vertical line indicates time of implementation of the See-and-Treat.

The intervention influenced the number of total and acute visits (Figs 1 and 2). Before implementation, there was a significant increase in total and acute visits: total visits/1000 listed patients increased by 7.0 per quarter (*P* = 0.037); acute visits/1000 listed patients increased by 3.0 per quarter (*P* = 0.019) during the year prior to implementation. One year after, the increase in total visits reversed to a trend towards reduction by 29.4 per quarter (*P* = 0.119), and the increase in acute visits reversed to a statistically significant reduction (reduced by 26.7 per quarter (*P* = 0.006)). Reduction in acute visits started the second quarter after implementation (−14.0 visits/1000 patients (*P* = 0.019)).

**Figure 3:**
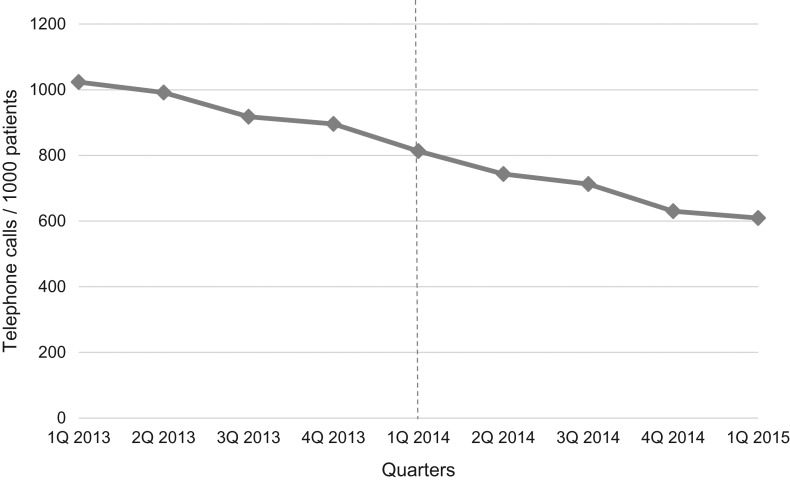
Number of telephone calls per 1000 listed patients from the first quarter of 2013 till the first quarter of 2015 (after adjustment for seasonality). Dotted vertical line indicates time of implementation of the See-and-Treat.

The year prior to implementation saw a significant reduction in calls (by 52.0 calls/1000 patients per quarter (*P* = 0.000)) (Fig. 3). The year after implementation, the trend towards reduction in calls continued, but did not reach statistical significance (by 21.4 calls/1000 patients per quarter (*P* = 0.265)).

**Figure 4:**
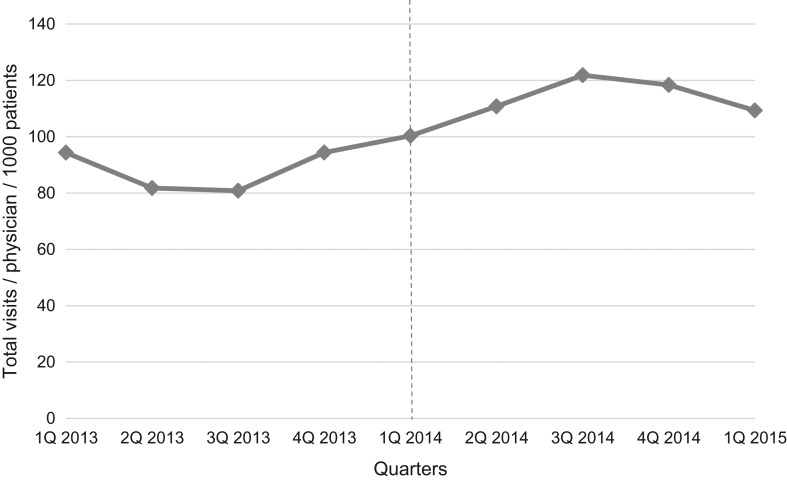
Total number of visits/physician per 1000 listed patients from the first quarter of 2013 to the first quarter of 2015 (after adjustment for seasonality). Dotted vertical line indicates time of implementation of the See-and-Treat.

Productivity did not increase (Fig. 4). Before implementation, there was a small and non-significant productivity increase (total number of visits/physician per 1000 listed patients by 2.5 per quarter (*P* = 0.352)). The year after implementation, productivity increased, but did not reach statistical significance (an increase of total number of visits/physician per 1000 listed patients by 9.6 per quarter (*P* = 0.570)). Observational data showed a reduction in time-per-patient of up to 88%.

#### Patient satisfaction

**Figure 5:**
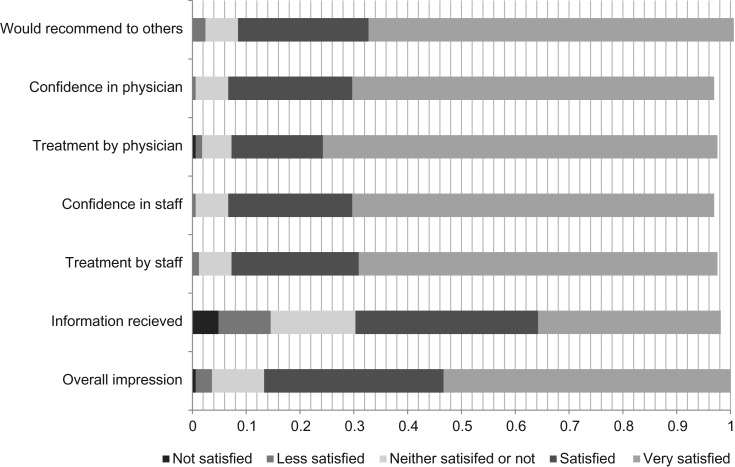
Results of the patient questionnaire.

The 289 questionnaires that were collected revealed that most of the patients were very satisfied or satisfied with the care experience (Fig. 5).

#### Staff experience

Staff perceived that process standardization and the symptom forms increased efficiency and improved quality. Standardizing the physical layout and the symptom forms sped up consultations. Symptom forms contributed to information accuracy, a patient focus, adherence of patients to the care plan and made it easier to quickly identify patients outside the See-and-Treat patient segment. Physicians described that an unexpected benefit of working in a standardized fashion was the increased ability to identify other pathological conditions. Merging payment and lab activities was described as a time-saver.

Staff enjoyed working efficiently and at a high pace. All physicians had several years of ED or trauma care experience and highlighted the ‘rush’ of helping many people quickly. LPNs felt satisfaction seeing patients express happiness about a smooth and efficient care experience.

The manager described that the increased productivity eventually led to a lowered reimbursement rate as the Fee-for-service had a predetermined ceiling negotiated annually. The manager did not see this as a reason to discontinue the project because the increased productivity meant that the concerns of more patients could be addressed and reliance on temporary physicians could be reduced. It also created an opportunity to renegotiate terms.

### Observed associations between outcomes, interventions and relevant contextual elements

Observations revealed inconsistencies, which could explain the non-significant increase in productivity. While all physicians discussed the importance of establishing optimal and standardized protocols, they often deviated from these, adding additional examinations based on personal preference and experience. We found several examples of ‘mission creep’ where limitations imposed by the symptom forms were ignored or overruled. Not only if the physician could resolve it quickly, e.g. sick-leave certificates, but even patients with chronic conditions were seldom redirected. One doctor worried that the See-and-Treat could thereby lose the very qualities that made it unique and instead become ‘a small PHCC within the PHCC’.

## Discussion

The translation of the See-and-Treat approach to primary care led to a significant decrease in acute visits and a decrease in a total number of visits. The pre-existing trend of diminishing telephone contacts continued. A non-significant increase in physician productivity was observed. Overall, patients were very satisfied and staff perceived improved efficiency and quality.

The reduction in visits and telephone contacts could indicate that, somewhat paradoxically, increasing access to primary care physicians does not ‘open the floodgates’ nor increase demand, as was reported for WIC and RC [4, 10]. Indeed, the introduction of access barriers, such as telephone triage, can increase care utilization [23]. A partial explanation could be that when care is readily available, the inclination to book appointments as precautionary measures against eventualities decreases. It remains to be seen how reducing mission creep or task-shifting to nurses could further improve outcomes.

Despite treating substantially more patients (up to 10 patients/hour instead of 4), the increase did not reach statistical significance. A partial explanation is the decreased use of temporarily employed physicians. Spending more time on patients with complex conditions or administrative tasks are both plausible explanations.

Nurse-driven triage systems can increase accessibility in primary care [24], but the use of patient-driven triage is novel. The symptom form directly involved patients in their care process and can be seen as a step towards more patient-driven co-care. Similar patient self-triage tools can be as accurate as traditional triage systems in EDs [25], and improve efficiency, quality, and reduce waiting times in other areas [26, 27]. The See-and-Treat approach had a positive impact for a specific segment of patients presenting with a predefined group of ‘acute’ complaints. These patients differ from those with conditions who may require coordination with other professions and services. Thus, the different sub-populations/segments that exist within primary care may benefit from specifically tailored approaches designed to meet their needs efficiently and effectively.

Our findings highlight the importance of partnering with patients and staff in the design and implementation of an intervention. Patient engagement moved beyond patients as passive recipients of care to collaborative partners in the diagnostic process. Through the LPN’s interaction with patients, it became clear that information to support awareness of the new service had to be continually improved. Staff were engaged in design and implementation from the start through analyses of the presenting complaints and variations in diagnostic practices in order to identify inclusion criteria and establish standardized diagnostic routines. This involvement coupled with managerial support most likely had a positive impact on empowering and engaging staff. Consequently, when questions were raised, such as about the quality of documentation practices—staff went on to develop better routines. It remains to be seen if personal interest in emergency care predisposes particular physicians for See-and-Treat. If so, interventions that help others see the value of investing resources and infrastructure for ‘simple cases’ may be important to support further dissemination.

### Limitations

Several measures were employed to strengthen reliability and internal validity. Interview and observation protocols guided data collection. Multiple data sources were triangulated and particular attention was paid to the interaction between context and intervention as recommended by SQUIRE 2.0.

External validity was limited by the single-case study design. However, the uniqueness of the See-and-Treat with patient self-triage made any other design difficult. Future studies could focus on translation of See-and-Treat to multiple PHCCs and include performance measures beyond volume and productivity, such as quality of care, costs and utilization of ED services.

We were limited to analyzing intervention effects on the PHCC’s acute visits as See-and-Treat patients were not identifiable in the administrative system. This could have diluted the effect of the intervention. It did hinder a more precise quality analysis in terms of health outcomes or through proxy measures such as return visits or visits to other care providers, such as EDs. The continued reduction in telephone contacts could have been influenced by a nationwide web-based health information platform roll-out. Information about non-responders would have strengthened the patient experience analysis. However, this was difficult to collect within the resource and logistical constraints of the study—symptomatic of the challenges of quality improvement research in clinical settings with high patient throughput.

## Conclusion

This study describes how a See-and-Treat process tailored to meet the needs of patients with less severe conditions in an efficient manner may be a new model for primary care to consider. As a first exploration of a See-and-Treat application in primary care, three essential differences from WIC and RC were identified:

- It is part of a primary care setting (not adjacent to a hospital, ED, nor in a pharmacy).
- It is staffed with physicians, not nurses.
- A patient self-triage tool is utilized that contributes to patient involvement and standardization of the care process.

These aspects most likely contributed to improved access by improving the efficiency and quality of the service. Seen within the context of a primary care center, more consistent use of the patient-triage system to limit which patients are seen and prevent mission creep, could increase physician productivity further. The use of digitalized process tools could also improve efficiency and quality, especially for documentation. Efficiency gains could be shifted to other patient segments, such as patients with chronic conditions, as well as strengthen the role primary care has in ensuring healthy lives and well-being for all at all ages, i.e. the third Sustainable Development Goal. The structured engagement of patients in self-triage could be translated to other contexts, such as EDs or for scheduled visits to outpatient specialist centers.
